# Bi-Att3DDet: Attention-Based Bi-Directional Fusion for Multi-Modal 3D Object Detection

**DOI:** 10.3390/s25030658

**Published:** 2025-01-23

**Authors:** Xu Gao, Yaqian Zhao, Yanan Wang, Jiandong Shang, Chunmin Zhang, Gang Wu

**Affiliations:** 1 School of Computer and Artificial Intelligence, Zhengzhou University, Zhengzhou 450001, China; gaoxu@zzu.edu.cn (X.G.); zyq15516640907@gs.zzu.edu.cn (Y.Z.); wangyanan991224@163.com (Y.W.); sjd@zzu.edu.cn (J.S.); 2 National Supercomputing Center in Zhengzhou, Zhengzhou 450001, China; 3 Yutong Bus Co., Ltd., Zhengzhou 450000, China; zhangcm@yutong.com

**Keywords:** multi-modal sensor fusion, attention mechanism, 3D object detection, autonomous driving

## Abstract

Currently, multi-modal 3D object detection methods have become a key area of research in the field of autonomous driving. Fusion is an essential factor affecting performance in multi-modal object detection. However, previous methods still suffer from the inability to effectively fuse features from LiDAR and RGB images, resulting in a low utilization rate of complementary information between depth and semantic texture features. At the same time, existing methods may not adequately capture the structural information in Region of Interest (RoI) features when extracting them. Structural information plays a crucial role in RoI features. It encompasses the position, size, and orientation of objects, as well as the relative positions and spatial relationships between objects. Its absence can result in false or missed detections. To solve the above problems, we propose a multi-modal sensor fusion network, Bi-Att3DDet, which mainly consists of a Self-Attentive RoI Feature Extraction module (SARoIFE) and a Feature Bidirectional Interactive Fusion module (FBIF). Specifically, SARoIFE captures the relationship between different positions in RoI features to obtain high-quality RoI features through the self-attention mechanism. SARoIFE prepares for the fusion stage. FBIF performs bidirectional interaction between LiDAR and pseudo RoI features to make full use of the complementary information. We perform comprehensive experiments on the KITTI dataset, and our method notably demonstrates a 1.55% improvement in the hard difficulty level and a 0.19% improvement in the mean Average Precision (mAP) metric on the test dataset.

## 1. Introduction

Autonomous driving requires an accurate and efficient perceptual model to portray complex driving environments. Three-dimensional object detection is one of the essential tasks in autonomous driving perception systems to provide critical object information for downstream tasks (e.g., semantic segmentation, object tracking, and path planning) [1,2]. Currently, some LiDAR-based methods [3,4,5,6] struggle to generate accurate 3D bounding boxes, limiting object detection performance due to the sparsity of point clouds and the poor quality of information in long-range and occluded regions. In contrast, RGB images provide rich semantic and texture information, but they lack the accurate depth information present in point clouds. To fully utilize the advantages of LiDAR and RGB images, various researchers have proposed some multi-modal approaches [7,8,9] to exploit complementary information to improve the accuracy of 3D object detection.

**Figure 1:**
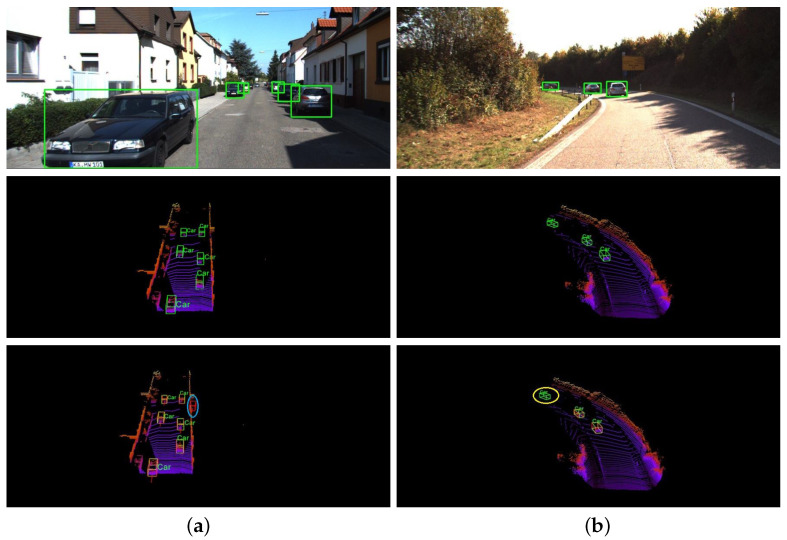
The existing methods have the problems of missing detection in complex scenes and the easy mis-detection of distant objects. The top two lines and bottom depict the ground truth and the detected results of SFD [19], respectively. The light blue ellipse shows the false detection and the yellow one gives the missed object. (**a**) False detection. (**b**) Missed detection.

Depending on the stage of fusion, multi-modal approaches can generally be categorized into three groups [2]: early fusion [10,11,12], intermediate fusion [3,13,14], and late fusion [15,16]. In particular, intermediate fusion is the common strategy adopted by most current models. Point/voxel level feature fusion [3,11] and RoI fusion [13,17] are two representative intermediate fusion strategies. Point/voxel feature fusion achieves complementary fusion at a more detailed level and is not limited by the RoI. However, the point cloud contains too many points, requiring excessive computation. In contrast, RoI fusion usually focuses on the RoI only, which can reduce the total computation. Meanwhile, RoI fusion, selected as our fusion strategy, incorporates a fine-grained regional attention mechanism to enhance focus on object details. The quality of RoI features is crucial in RoI fusion for the object detection task. It directly affects the localization, recognition and understanding of the object in RoI. A series of previous methods [3,5] have argued that point features could offer more positional information, leading to more accurate object detection results. However, SARFE [18] demonstrated that accurate point information leads to performance degradation. While point features excel in providing positional accuracy, the absence of structural information may lead to missed and false detections, as shown in Figure 1. Rich structural information facilitates the network to infer the position and shape (size and scale) of objects, especially for 3D objects with relatively fixed structures. Simultaneously, by capturing the internal structure of the object and the associations with other objects, the rich structural information enables our network to better understand the relationship between the objects and their surroundings.

**Figure 2:**
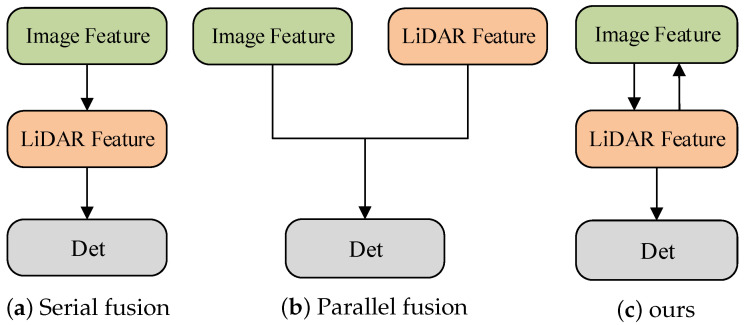
LiDAR–camera fusion strategy. (**a**) Serial fusion: limited by LiDAR sensors. (**b**) Parallel fusion: dual streams work independently. (**c**) Bidirectional interactive fusion (ours): intermodal bidirectional interactions make full use of complementary information.

As shown in Figure 2, the existing fusion models primarily have two forms: serial fusion [7,20,21] and parallel fusion [19,22,23,24]. While serial fusion methods can enhance the point cloud representation, LiDAR signals are often influenced by various conditions such as weather, lighting, and reflections. They are inherently over-dependent on the LiDAR modal without exploring in depth the importance of RGB images for detecting objects. It fails to fully utilize the rich semantic information from camera sensors and cannot adequately predict when LiDAR signals fail. On the other hand, the parallel fusion methods alleviate the serial fusion methods’ dependence on LiDAR and introduce two modalities to enhance detection accuracy. However, most current parallel fusion methods rely on a single modality to guide the other modality, which restricts information flow. Specifically, many methods take the features extracted from point cloud data as the primary modality and the image features as the auxiliary modality, typically fusing the auxiliary modality’s features with the primary modality through concatenation or query-based approaches. This approach results in an unbalanced information exchange. They do not consider that bidirectional interactions between the two modalities can better exploit their complementarity. Consequently, the correlation between the depth information of the point cloud and the dense semantic information of the RGB images may be limited.

To address such problems, this paper proposes a multi-modal fusion framework called Bi-Att3DDet. The framework consists of two main parts: the self-attentive RoI feature extraction module (SARoIFE) and the Feature Bidirectional Interactive Fusion module (FBIF). The existing multi-modal methods such as SFD [19] focus on the feature extractor (CPConv) in the pseudo point cloud branch and the RoI fusion strategy (3D Grid-wise Attentive Fusion). It uses CPConv to explore both 2D image features and 3D geometric features of the pseudo point cloud, and the RoI fusion strategy combines the original and pseudo RoI features. However, as mentioned earlier, this fusion strategy follows single-modal guidance for the other modality, which limits the sufficient interaction of information. In our fusion framework, SARoIFE is mainly applied to the raw point cloud and uses a self-attention mechanism to dynamically adjust weights to balance different local features. FBIF performs bidirectional interaction between the raw LiDAR and the pseudo RoI features to fully leverage complementary information. At the same time, the interacted features are fused using a simple attention mechanism, and the fused RoI features are used for further box refinement. Finally, the effectiveness of our approach is demonstrated through extensive experiments on the widely used KITTI dataset. In summary, our contributions are as follows:

1. We introduce a self-attentive RoI feature extraction module, SARoIFE, in the raw point cloud branch, employing an attention mechanism to balance different local features to obtain high-quality RoI features.
2. We propose a novel Feature Bidirectional Interactive Fusion module, FBIF, aiming to fully leverage the complementary information between the two modalities of the raw and pseudo RoI features through a bidirectional interaction manner.
3. Extensive experiments validate the effectiveness of our method, demonstrating superior performance on the KITTI 3D object detection dataset compared to others.

## 2. Related Works

### 2.1. Three-Dimensional Object Detection Based on a Single Sensor

Current commonly used single-sensor 3D object detection methods are typically categorized into LiDAR-only [4,25,26,27] and camera-only [28,29,30]. In the past, most researchers were very interested in the latter methods. Specifically, CaDDN [28] designs a Frustum feature network to convert RGB images into 3D space to generate bird’s-eye-view (BEV) representations. Pseudo-LiDAR [29] uses stereo images to obtain depth information and generate pseudo points for 3D object detection. PGD [30] builds a graph of geometric relationships between predicted objects and uses this graph to facilitate depth estimation. While cameras are commonly utilized sensors, they often lack depth information to achieve accurate detection. In recent years, LiDAR-based methods have become the focus of extensive research. Voxel-based [4,25] and point-based [26,27] methods have received significant attention. Although point-based methods have been praised for their accurate detection and the utilization of information to enhance accuracy, the process of sampling and feature aggregation from irregular point clouds is time-consuming. Additionally, the extracted structural information of point cloud features is limited. Therefore, many researchers use voxel-based methods to discretize the unordered point cloud spaces into regular voxel grids, achieving a balance between efficiency and accuracy through sparse convolution. VoxelNet [25] introduces a sparse voxel grid and a novel VFE layer for point feature extraction. Voxel-RCNN [4] presents a simple yet effective voxel-based framework by leveraging voxel features in a two-stage approach. Although LiDAR-based methods offer accurate depth information, the captured point clouds are often irregular, sparse, and sensitive to weather conditions.

### 2.2. Three-Dimensional Object Detection Based on Multiple Sensors

Due to the limitations in LiDAR and camera sensors, the fusion of LiDAR and camera features has recently received a lot of attention. MV3D [31] is a pioneering work in multi-modal 3D object detection using multi-view aggregation. AVOD [13] integrates LiDAR BEV features with camera features, feeding the fused data into a Region Proposal Network (RPN) to generate 3D bounding boxes. Meanwhile, 3D-CVF [32] adopts a cross-view spatial feature fusion strategy to fuse extracted LiDAR features with camera features. EPNet [14] introduces an LI-Fusion module to enhance point features by integrating semantic features at the point level. Building upon EPNet, EPNet++ [33] devises an IF-Fusion module for the cascaded bidirectional fusion of LiDAR and camera features. Inspired by it, this paper proposes a Feature Bidirectional Interactive Fusion module.

Previous methods projected 3D point clouds onto a 2D plane suffering a mismatch between the resolution of the point cloud and RGB images, resulting in a loss of 3D spatial information. Recently, many methods have addressed this challenge by utilizing virtual (pseudo) points to lift RGB images from 2D to 3D space. MMF [34] converts the depth map into pseudo lidar points through depth estimation, aiming at the learning of multiple related tasks. MVP [35] generates dense 3D virtual (pseudo) points from the 2D detection results and combines the generated virtual (pseudo) points with the raw point cloud for more accurate localization and recognition. VPFNet [36] aligns and aggregates point cloud and image data using virtual (pseudo) points as multi-modal data fusion locations, thereby preventing information loss. Similarly, SFD [19] transforms 2D images into 3D virtual (pseudo) point clouds via deep completion, bridging the heterogeneity gap between data representations. Virconv [12] introduces the VirConvNet convolutional operator, leveraging an early fusion to fuse raw and virtual (pseudo) point clouds, thereby addressing noise and redundant computation issues associated with generating virtual (pseudo) point clouds. Most methods, due to the unidirectional guidance between modalities during fusion, lead to an underutilization of the richness of multi-modal information.

### 2.3. Attention Mechanism in Vision

The attention mechanism initially found widespread use in natural language processing tasks, and its application expanded with advancements in object detection. ViT [37], DETR [38], and Deformable DETR [39] integrate the attention mechanism with CNNs for 2D object detection tasks. Subsequently, DETR3D [40] extends this approach to 3D object detection. The evolution of the attention mechanism has introduced new opportunities for multi-modal fusion methods. With attention-based approaches, networks can learn weight distributions, assigning varying weights to different parts of input data or feature maps. This enables the retention of crucial information while disregarding irrelevant data. Additionally, 3D-CVF [32] employs an adaptive gated fusion network to merge feature maps. DeepFusion [41] introduces a LearnableAlign, which utilizes cross-attention to capture correlations between images and LiDAR features during fusion. LoGoNet [42] presents a novel local-to-global fusion network that leverages a feature dynamic aggregation module to facilitate information interaction between local and global features for improving detection performance. DyFusion [43] proposes a cross-attention dynamic fusion to merge heterogeneous data.

## 3. Proposed Approach

**Figure 3:**
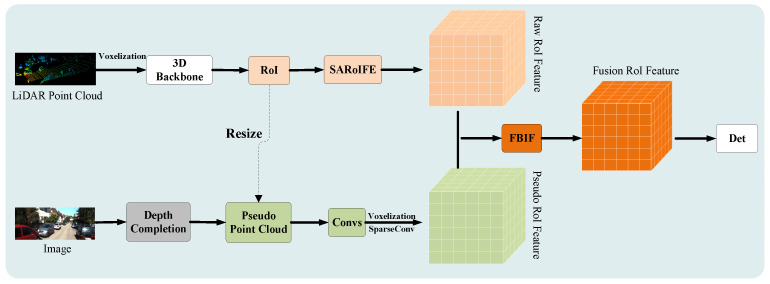
Bi-Att3DDet framework.

In this section, we give a multi-modal fusion framework, Bi-Att3DDet, as illustrated in Figure 3. By incorporating data from different sensors (LiDAR and camera) as inputs, Bi-Att3DDet generates the corresponding RoI features through a dual-branch structure. Our framework focuses on the structural information of local features in the raw point cloud branch using the proposed SARoIFE module (Section 3.1). This module employs multiple self-attentive modules in series to dynamically weigh diverse local features, resulting in high-quality raw RoI features. In the image branch, the framework operates similarly to Virconv [12], lifting the image from 2D to 3D through depth completion. It utilizes CPConvs [19] to extract rich information from pseudo points, thereby generating pseudo RoI features. The FBIF module (Section 3.2) employs cross-attention and adaptive fusion to enable inter-modal bidirectional interaction and the comprehensive integration of information. Each proposed component is described in the following subsections.

### 3.1. Self-Attentive RoI Feature Extraction

**Figure 4:**
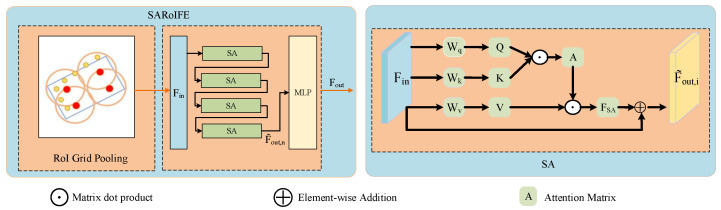
Illustration of self-attentive RoI feature extraction.

In 3D object detection, structural information encompasses the position, size, orientation, and spatial relationships of objects, which are essential for determining their 3D coordinates and understanding their geometric structure. The self-attentive RoI feature extraction module, SARoIFE, as shown in Figure 4, utilizes RoI grid pooling to focus on local features. However, when features extracted from each grid point exhibit high similarity, this similarity can obscure the local feature structure in RoI. For instance, objects with similar textures or appearances across different locations or scales may result in similar representations in the feature space. As a result, the detailed information on local features may be blurred, impairing the network’s ability to distinguish them effectively.

Self-attention is a widely used feature extraction mechanism in natural language processing and computer vision. Its core idea is to dynamically assign attention by computing the correlation weights between different elements within the data. SARoIFE utilizes this self-attention mechanism to learn the relationship matrix between points and adaptively assigns weights to different features, enabling key features to receive more attention. This capability is particularly beneficial for point cloud data, which are often sparse and characterized by unevenly distributed critical features. Additionally, the unorderedness of point cloud data poses challenges for traditional methods that require fixed ordering or sorting. In contrast, self-attention is inherently order-invariant and can directly process unordered point clouds. Furthermore, stacking multiple layers of self-attention progressively enhances the representation of high-dimensional features, leading to more robust feature extraction with enriched global semantic information and structural information.

Specifically, the features denoted as $F_{in}\in R^{N\times d_{i}}$ obtained from RoI grid pooling are fed into the SA module. Here, *N* is the total number of grids in a 3D RoI, and $d_{i}$ is the grid feature channel. Following the terminology in attention is all you need [44]; we define the query matrix ($Q_{i}$), key matrix ($K_{i}$), and value matrix ($V_{i}$), which are generated through linear transformations from $F_{in}\in R^{N\times d_{i}}$, as shown in Equation (1):

(1) $$\begin{array}{c} \left(Q_{i},K_{i},V_{i}\right)=\left\{\begin{array}{cc} F_{in}\cdot \left(W_{q}^{\left(i\right)},W_{k}^{\left(i\right)},W_{v}^{\left(i\right)}\right), & i=1,\\ {\tilde{F}}_{\mathrm{out},i-1}\cdot \left(W_{q}^{\left(i\right)},W_{k}^{\left(i\right)},W_{v}^{\left(i\right)}\right), & i=2,\cdots ,n. \end{array}\right. \end{array}$$

where $W_{q}^{\left(i\right)}$, $W_{k}^{\left(i\right)}$, and $W_{v}^{\left(i\right)}$ represent the shared learnable linear transformations, $i\in Z^{+}$ stands for the *i*-th SA block, and *n* is the number of SA blocks. $Q_{i},K_{i}\in R^{N\times d_{a}},V_{i}\in R^{N\times d_{i}}$, and $d_{a}\in Z^{+}$ denote the dimensions of the query and key matrices, and $d_{i}\in Z^{+}$ indicates the dimension of the value matrix.

Following the derivation of $Q_{i}$, $K_{i}$, and $V_{i}$, we use the query and key matrices to perform matrix dot product computations and normalization to obtain the attention weight $A_{i}$:

(2) $$A_{i}=\mathrm{softmax}\left(\frac{Q_{i}{K_{i}}^{T}}{\sqrt{d_{a}}}\right)$$

The self-attention output feature $F_{SA,i}$ is computed by Equation (3). We use the calculated attention weights $A_{i}$ to perform a weighted sum of the value vectors $V_{i}$.

(3) $$F_{SA,i}=A_{i}\cdot V_{i}$$

Then, we obtain the output feature ${\tilde{F}}_{out}$ of the self-attention modules via residual concatenation. The self-attentive RoI feature extraction module consists of *n* self-attentive modules connected in series, with the output denoted as ${\tilde{F}}_{out,i}$, where i=1,⋯,n. In the *i*-th layer, the SA module takes the output feature ${\tilde{F}}_{\mathrm{out},i-1}$ from the previous layer as input to generate the current layer’s output feature ${\tilde{F}}_{\mathrm{out},i}$, as in Equation (4). Finally, the output features ${\tilde{F}}_{\mathrm{out},n}$ are followed by a fully connected layer to obtain the enhanced RoI features $F_{out}$, calculated as Equation (5):

(4) $$\begin{array}{cc} {\tilde{F}}_{out,i}=\mathrm{ReLU}\left(\mathrm{BN}\left(\mathrm{MLP}\left(F_{SA,i}\right)\right)\right)+F_{in},\; & i=1,\cdots ,n \end{array}$$

(5) $$\begin{array}{c} F_{out}=\mathrm{MLP}\left({\tilde{F}}_{out,n}\right) \end{array}$$

### 3.2. Feature Bidirectional Interactive Fusion

**Figure 5:**
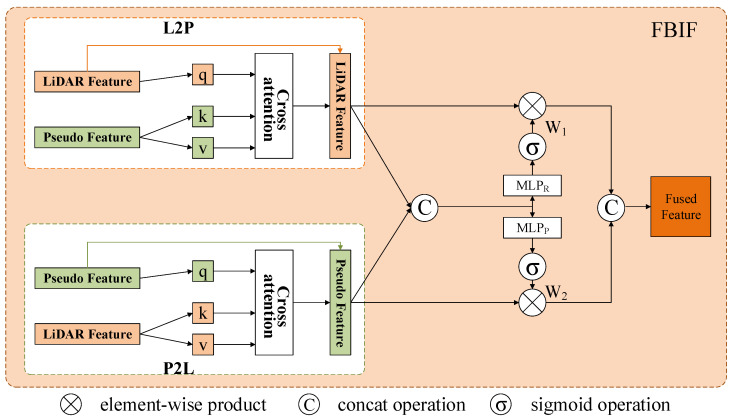
Illustration of Feature Bidirectional Interactive Fusion.

The raw RoI features and pseudo RoI features obtained from feature extraction need to be fused to enhance detection performance. However, existing fusion methods [20,41] often involve simple serial connections or a single modality guiding the other, leading to unidirectional information flow and inadequate fusion. Benefiting from the idea of bidirectional fusion in EPNet++ [33], we design a Feature Bidirectional Interactive Fusion (FBIF) module to facilitate bidirectional fusion between the two modalities. However, we use cross-attention for inter-modal interaction, as shown in Figure 5.

FBIF enables the mutual perception of two modal features, selectively combining the RoI features using an attention mechanism to achieve feature integration from different sources. We use the cross-attention mechanism to aggregate the complementary information. In the cross-attention mechanism, features from one modality are matched with those from another modality through queries, thereby transferring attention information and enabling information interaction and fusion. For example, LiDAR data provide spatial geometric information, while RGB images offer color and texture information. Through the cross-attention mechanism, features from both modalities can interact efficiently, fully utilizing the unique information from each modality to achieve a more comprehensive feature representation. Moreover, when interacting with another modality, the cross-attention mechanism adaptively assigns different weights to each feature by calculating the similarity between queries and keys. This dynamic weighting helps the model automatically learn the importance of features from different modalities, thereby improving the quality of the fused features.

We define the LiDAR RoI feature as $F_{L}\in R^{N\times d_{k}}$ and the pseudo RoI feature as $F_{P}\in R^{N\times d_{k}}$, where $d_{k}\in Z^{+}$ denotes the dimension of the features. To this end, we conduct Feature Bidirectional Interaction using the L2P and P2L branches, respectively.

L2P. For this branch, we use LiDAR RoI features as queries to guide the pseudo RoI features, with the pseudo RoI features serving as keys and values. The dynamic correlation between the two modalities is captured through a multi-head cross-attention mechanism with *m* heads. Specifically, the input contains LiDAR RoI features $F_{L}\in R^{N\times d_{k}}$, along with all corresponding pseudo RoI features $F_{P}\in R^{N\times d_{k}}$. The LiDAR RoI features are converted into queries $Q_{i}$ and the pseudo RoI features are converted into keys $K_{i}$ and values $V_{i}$ using three separate fully connected layers. For each query $Q_{i}$, we perform an inner product between the query $Q_{i}$ and the key $K_{i}$ to obtain the attentional affinity matrix. Subsequently, we employ the attentional affinity matrix to perform a weighted aggregation of $V_{i}$, obtaining the features from the *i*-th head, as in Equation (6). Finally, we concatenate the features from the $m\in Z^{+}$ head and connect them with the raw LiDAR features $F_{L}$ to obtain the interacting features $F_{LP}$. Its implementation is shown in Equation (7):

(6) $${\mathrm{head}}_{i}=\mathrm{softmax}\left(\frac{Q_{i}K_{i}^{T}}{\sqrt{d_{k}}}\right)V_{i}$$

(7) $$F_{LP}=\mathrm{Concat}\left({\mathrm{head}}_{1},{\mathrm{head}}_{2},\ldots ,{\mathrm{head}}_{m}\right)+F_{L}$$

P2L. This branch is similar to the L2P branch, with the distinction in the guidance objects. Here, the pseudo RoI features guide the search for relevant information within the LiDAR RoI features, through the pseudo RoI features serving as queries and the LiDAR RoI features as the keys and values. This branch enables us to obtain the interacted features $F_{PL}$. Leveraging the multi-head cross-attention mechanism enhances the model’s capacity to process multi-source data, facilitating a more comprehensive understanding and integrating information across different modalities.

To combine the interactive features $F_{LP}$ and $F_{PL}$, we employ an adaptive fusion network to select features conducive to detection. Initially, we feed the interactive features $F_{LP}$ and $F_{PL}$ into a fully connected layer and concatenate the outputs to form a compact feature representation. Subsequently, a pair of weights ($w_{1}$ and $w_{2}$) is obtained through fully connected layers followed by a sigmoid operation, as in Equations (8) and (9). The sigmoid operation constrains the weights $w_{1}$ and $w_{2}$ within the range of 0 to 1, enabling the network to adaptively adjust the contribution of each modality based on the significance of the current features. During network training, $w_{1}$ and $w_{2}$ are dynamically optimized through the loss function and backpropagation. By learning the distribution patterns of features in different input scenarios, the network adaptively generates weights, allowing it to assign the optimal weighting ratio for each specific input during inference. This approach overcomes the limitations of manually setting fixed weights. The generated weights $w_{1}$ and $w_{2}$ are applied to the features $F_{LP}$ and $F_{PL}$ through element-wise multiplication. Specifically, $w_{1}$ is applied to $F_{LP}$, and $w_{2}$ is applied to $F_{PL}$, as in Equations (8) and (9). This weighting approach ensures that the features from different modalities are adjusted according to the dynamically generated weights, achieving effective integration of information from both modalities. Finally, these weighted features are fused through concatenation to obtain the final fused feature $F_{f}$, which is calculated as Equation (10):

(8) $$\begin{array}{cc} F_{LP} & =F_{LP}\times \underset{\mathrm{Raw}\;\mathrm{LiDAR}\;\mathrm{Attention}\;\mathrm{Map}}{\underset{︸}{\sigma ({\mathrm{MLP}}_{R}\left(F_{LP}⊕F_{PL}\right))}} \end{array}$$

(9) $$\begin{array}{cc} F_{PL} & =F_{PL}\times \underset{\mathrm{Pseudo}\;\mathrm{LiDAR}\;\mathrm{Attention}\;\mathrm{Map}}{\underset{︸}{\sigma ({\mathrm{MLP}}_{P}\left(F_{LP}⊕F_{PL}\right))}} \end{array}$$

(10) $$\begin{array}{cc} F_{f} & =\mathrm{CONCAT}(F_{LP},F_{PL}) \end{array}$$

where × denotes the element-wise product operation, and ⊕ represents the channel-wise concatenation operation. $MLP_{R}$ and $MLP_{P}$ are fully connected layers, and σ represents the sigmoid function.

### 3.3. Loss Function

We implement the loss function based on Voxel-RCNN [4]. The Region Proposal Network loss and the refinement network loss are denoted as $L_{rpn}$ and $L_{rcnn}$, respectively. The total loss can be expressed by Equation (11):

(11) $$L_{total}=L_{rpn}+L_{rcnn}$$

Following SECOND [45], we design the loss of RPN as a combination of classification loss and bounding box regression loss, formulated as Equation (12):

(12) $$L_{\mathrm{rpn}}=\alpha_{1}L_{\mathrm{cls}}+\alpha_{2}L_{reg}$$

where $\alpha_{1}$ and $\alpha_{2}$ are set to 1 and 2, respectively. We adopt the focus loss [46] as $L_{\mathrm{cls}}$ to balance positive and negative samples in the categorization loss while employing the ${smooth}_{L1}$ loss as $L_{reg}$ for bounding box regression. The refinement network loss $L_{rcnn}$ consists of the Intersection over Union (IoU) confidence prediction loss $L_{iou}$ and the 3D bounding box refinement loss $L_{refine}$ as (13):

(13) $$L_{\mathrm{rcnn}}=L_{\mathrm{iou}}+L_{\mathrm{refine}}$$

## 4. Experiments

### 4.1. Dataset and Evaluation Metrics

We evaluate our approach on the KITTI 3D object detection benchmark. The KITTI dataset [47] comprises 7481 LiDAR and image frames for training and 7518 frames for testing. The objects are categorized into three difficulty levels, easy, moderate, and hard, based on the size of the object, the degree of occlusion, and the degree of truncation. For experimental validation, we split 7481 training samples into a training set of 3712 frames and a validation set of 3769 frames. To evaluate the results, we use the widely used metrics: 3D Average Precision (AP) under 40 recall thresholds and 3D Average Precision under 11 recall thresholds. The IoU thresholds for cars, pedestrians, and cyclists are 0.7, 0.5, and 0.5, respectively.

### 4.2. Implementation Details

Network Settings. In our method, virtual (pseudo) points are generated following the approach described in TWISE [48]. We set the range of the point cloud on the (x,y,z) axis as [0,70.4] m, [−40,40] m, and [−3,1] m, respectively. The voxel structure is divided by voxel size (0.05,0.05,0.1) m, with a maximum of five points per voxel. For the self-attentive RoI feature extraction, we follow the common settings used in VoxelRCNN [4]. RoI pooling is performed using a 6 × 6 × 6 grid, with RoI pooling adjacency radius of 0.4 m, 0.8 m, and 1.6 m, respectively. Additionally, the self-attentive RoI feature extraction module consists of *n* = 4 self-attentive modules in series. In the Feature Bidirectional Interactive Fusion module, we set the number of attention heads to 4.

Training and inference details. To evaluate the efficiency of Bi-Att3DDet, we train it on a single 3090 GPU utilizing the ADAM optimizer, with a batch size of 4. We utilize a learning rate of 0.001 and a single-cycle learning rate strategy, incorporating a weight decay of 0.01 and a momentum of 0.9. The Bi-Att3DDet is trained for 40 epochs. For data augmentation during training, we employ ground-truth sampling, local rotation and translation, and global transformations. During training, we utilized a Non-Maximum Suppression threshold of 0.8 to generate 128 target proposals with a balanced ratio of negative and positive samples set at 1:1. For testing, we used a Non-Maximum Suppression threshold of 0.1 to remove redundant boxes.

### 4.3. Main Results

Results on the KITTI test set. We conduct a comparative analysis of our method with other approaches by submitting our results to the KITTI server for evaluation. Specifically, we compare our method with existing multi-modal approaches and LiDAR approaches. Table 1 presents a detailed quantitative comparison of various 3D object detection methods on the KITTI test set, with average precision over 40 recall positions (R40) used as the evaluation metric. This includes three difficulty levels: easy, moderate, and hard. Our method demonstrates convincing results when compared to existing LiDAR approaches, outperforming them across all three difficulty levels. This highlights the robustness and effectiveness of our approach. In the multi-modal approaches, although our proposed method lags behind the top-performing method (SFD) at the easy level, it demonstrates competitive performance at the moderate level. Notably, at the hard difficulty level, our method shows a significant improvement of 1.55% compared to SFD. Additionally, our approach achieves a 0.19% improvement in the mAP metric.

Results on KITTI val set. We present the results of car detection on the KITTI validation set in Table 2. Our method achieves better performance than the state-of-the-art method at three difficulty levels of cars. Specifically, it attains a high mAP of 90.49% on the KITTI validation set, with scores of 95.83%, 89.10%, and 86.53% for the easy, moderate, and hard levels, respectively. The best detection accuracy in each category is highlighted in bold.

Furthermore, our method proves highly effectiv for Car BEV detection, achieving an accuracy of 96.71%, 92.62%, and 92.13% for the easy, moderate, and hard levels, respectively. These results indicate gains of 0.47%, 0.53%, and 0.81% over the state-of-the-art method (SFD), as detailed in Table 3. Furthermore, we report the performance of our method using 3D AP based on 11 recall thresholds (R11) in Table 4. The results show improvements of 0.36%, 0.77%, and 0.93% over the SFD for the easy, moderate, and hard difficulty levels, respectively. Additionally, our method achieved an mAP accuracy of 88.04%. The results described above show that our method exhibits superior performance on various evaluation metrics, significantly improving upon existing methods in car detection tasks on the KITTI validation set.

### 4.4. Ablation Study

We perform ablation experiments on the KITTI validation set to evaluate the effectiveness of each component of our proposed method.

Effect of SARoIFE. The self-attentive RoI feature extraction module uses the attention mechanism to weight different local features within the proposal. By enhancing the structural information of various local features, SARoIFE generates high-quality RoI features. In addition, we use a four-layer SA in this module. In Table 6, we compare the detection results of the baseline model (Experiment (a)) and the baseline model enhanced with our proposed SARoIFE method (Experiment (b)). Experiment (a)’s results at the easy, moderate, and hard difficulty levels were 95.47%, 88.56%, and 85.74%, respectively. By incorporating SARoIFE into the baseline model, experiment (b) further extracted structural information at the raw point cloud branch. The results of experiment (b) at three different difficulty levels were 95.74%, 88.79%, and 86.04%, respectively. We observe that SARoIFE brought performance improvements of 0.27%, 0.23%, and 0.3% for all levels. This shows that SARoIFE contributes at three difficulty levels. Additionally, on the mAP metric, the performance of experiment (b) shows a 0.27% improvement compared to the baseline.

We conducted further experiments to verify the effectiveness of setting the parameter to *n* = 4 layers of SA in the SARoIFE module. As shown in Table 5, we tested different values for *n* to determine the optimal configuration. With three and five layers of SA, the detection performance of our method gains 0.02% and 0.06% in the mAP metric, respectively. With *n* = 4, the performance reaches 95.74%, 88.79%, and 86.03% for the easy, moderate, and hard levels, respectively. The mAP achieves 90.19%, showing the highest gain of 0.27%. These results indicate that setting *n* = 4 in the SARoIFE module yields the best performance at most metrics.

Effect of FBIF. As shown in Table 6, we add the proposed FBIF module based on experiment (b), which helps the raw RoI features and pseudo RoI features fully utilize complementary information in a bidirectional fusion manner during the feature fusion stage. Experiment (c) demonstrates the detection results for the three difficulty levels after adding the FBIF module: 95.83%, 89.10%, and 86.53%. This shows an improvement of 0.09%, 0.31%, and 0.49%. In addition, the FBIF shows a 0.3% improvement in the mAP metric. Based on the boosts in different difficulty levels, we believe that the FBIF module contributes more significantly to the hard and moderate levels. This is because the features in the pseudo point cloud provide a more effective complement to those raw point cloud features, especially in long-distance and complex scenes.

Multi-class performance. We further train the multi-class model to detect cars, pedestrians, and cyclists using a single model. Table 7 presents the results of 3D object detection for these multi-class scenarios, compared with the multi-class method SFD [19]. The multi-class model demonstrates significant performance gains in the pedestrian and cyclist categories. Our method shows significant improvements in the pedestrian category at the easy and moderate difficulty levels, achieving scores of 74.22% and 67.57%, respectively, which are 1.28% and 0.88% higher than SFD. For BEV detection, our method attains scores of 77.78%, 71.26%, and 65.74%. This is a great enhancement compared to SFD. In the cyclist category, our multi-class model shows remarkable performance improvements across all difficulty levels. Specifically, it achieves 95.46%, 73.60%, and 68.90% for the easy, moderate, and hard levels, respectively, marking increases of 2.07%, 0.65%, and 1.64% compared to SFD. Additionally, our method attains 95.86%, 76.06%, and 71.45% for BEV detection. The results show that our model can be extended to multi-class detection, exhibiting notable improvements, particularly for cyclists.

To further analyze why our proposed method outperforms the comparison methods in the category of cyclist, we conducted ablation experiments to verify the contribution of the proposed modules (SARoIFE and FBIF) to model performance. The results are summarized in Table 8. The performance at various levels is compared based on the R40 metric. The results demonstrate that the FBIF module makes the most significant contribution to performance improvement in the cyclist category. By dynamically balancing the feature contributions from different modalities, the FBIF module efficiently extracts complementary information across modalities. Furthermore, leveraging a parallel bidirectional feature interaction mechanism, the FBIF module integrates spatial and semantic information more comprehensively, enabling the more precise detection of small or geometrically complex objects in challenging scenarios.

**Figure 6:**
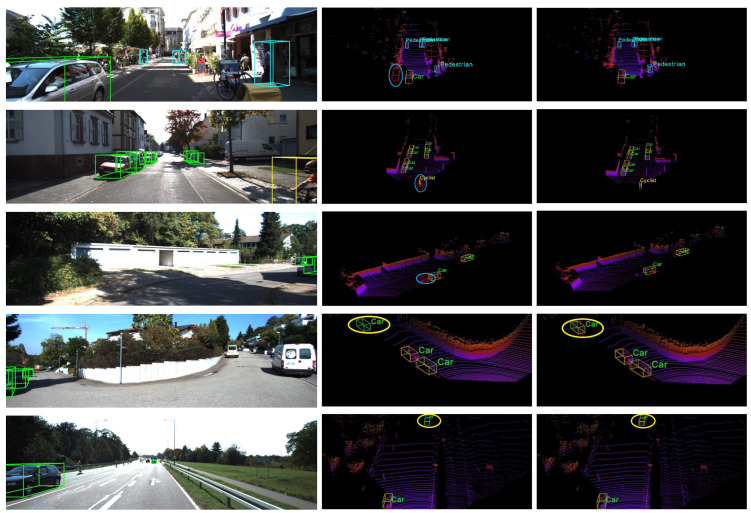
Comparison of visualization results between our method and theSFD method on KITTI. These images are the ground truth, SFD result, and Bi-Att3DDet (ours) result from left to right; the first three rows are false detection and the last two are missed detection in order from top to bottom. In the 3D detection result, the green box is the ground truth box and the red box is the prediction box. The blue circles denote false positives, and the yellow circles indicate undetected objects.

Qualitative Results. Figure 6 shows a visualization of the results predicted by our method and SFD [19], illustrating the effectiveness of our approach. Our method demonstrates accurate object detection across various scenarios, including long-range and complex object scenarios. Moreover, our method exhibits a reduced occurrence of misdetection phenomena compared to SFD [19], such as misidentifying objects with shadows or shapes similar to cars as actual cars.

## 5. Conclusions

The performance of 3D object detection tasks plays a crucial role in the safety of autonomous driving. To improve the accuracy of 3D object detection, we propose a multi-modal fusion framework called Bi-Att3DDet. In this framework, we focus on the structural information extraction in the raw point cloud branch and the feature fusion during the fusion stage. To effectively extract the structural information of points within RoI, we designed a self-attention RoI feature extraction (SARoIFE) module that enhances the structural information of local features using the self-attention mechanism and residual connections. It alleviates the loss of local feature structure within the RoI due to high similarity when extracting features from grid points. Additionally, in the fusion stage, considering that previous methods lack the full utilization of information between the two modalities (LiDAR and camera), we presented a feature bidirectional interaction fusion module. This module fuses the RoI features extracted from the raw and pseudo point cloud branch in a bidirectional interaction manner, thus fully utilizing the complementary information between modalities. To validate the detection performance of our model, we compare it with state-of-the-art methods on the KITTI set. Extensive experiments on the KITTI dataset demonstrate the effectiveness of Bi-Att3DDet. Moreover, we conducted a series of ablation experiments on the KITTI val set to prove these components’ effectiveness. Although our method outperforms other state-of-the-art methods in most metrics, there are still some limitations in complexity and detection accuracy. We will continue to improve the detection performance of the model and reduce its complexity in future efforts.

## Figures and Tables

**Table 1:** Car 3D detection results on the KITTI test set with R40. Bold represents the best performance.

Method	Reference	Modality	$3D_{R40}$			
			mAP	Easy	Mod.	Hard
SA-SSD [5]	CVPR 2020	LiDAR	80.90	88.75	79.79	74.16
CT3D [49]	ICCV 2021	LiDAR	82.25	87.83	81.77	77.16
VoxSeT [50]	CVPR 2022	LiDAR	82.68	88.53	82.06	77.46
FARP-Net [51]	TMM 2023	LiDAR	82.96	88.36	81.53	78.98
Voxel R-CNN [4]	AAAI 2021	LiDAR	83.19	90.90	81.62	77.06
PVT-SSD [52]	CVPR 2023	LiDAR	83.26	90.65	82.29	76.85
OcTr [53]	CVPR 2023	LiDAR	83.76	90.88	82.64	77.77
3D HANet [54]	TGRS 2023	LiDAR	84.18	90.79	84.18	77.57
GLENet [55]	IJCV 2023	LiDAR	84.44	91.67	83.23	78.43
EPNet [14]	ECCV 2020	LiDAR + RGB	81.23	89.81	79.28	74.59
VoPiFNet [56]	TITS 2024	LiDAR + RGB	82.07	88.51	80.97	76.74
Fast-CLOCs [16]	WACV 2022	LiDAR + RGB	82.15	89.10	80.35	76.99
CAT-Det [57]	CVPR 2022	LiDAR + RGB	82.62	89.87	81.32	76.68
HMFI [58]	ECCV 2022	LiDAR + RGB	82.71	88.90	81.93	77.30
CFPC [59]	TVT 2024	LiDAR + RGB	82.81	89.04	81.97	77.42
GraR-Pi [60]	ECCV 2022	LiDAR + RGB	83.45	90.94	82.42	77.00
DVF-PV [61]	WACV 2023	LiDAR + RGB	83.59	90.99	82.40	77.37
PA3DNet [62]	TII 2023	LiDAR + RGB	83.65	90.49	82.57	77.88
VPFNet [36]	TMM 2022	LiDAR + RGB	84.14	91.02	83.21	78.20
SQD [63]	ACM MM 2024	LiDAR + RGB	84.16	91.58	81.82	79.07
SFD [19]	CVPR 2022	LiDAR + RGB	84.80	**91.73**	**84.76**	77.92
Bi-Att3DDet (Ours)	-	LiDAR + RGB	**84.99**	91.03	84.46	**79.47**

**Table 2:** Car 3D detection results on the KITTI val set with R40. Bold represents the best performance.

Method	Modality	$3D_{R40}$			
		mAP	Easy	Mod.	Hard
Voxel-RCNN [4]	LiDAR	86.84	92.38	85.29	82.86
DVF-PV [61]	LiDAR	87.35	93.07	85.84	83.13
SE-SSD [64]	LiDAR	87.54	93.19	86.12	83.31
BtcDet [65]	LiDAR	87.76	93.15	86.28	83.86
CasA [66]	LiDAR	87.84	93.21	86.37	83.93
Graph-Po [60]	LiDAR	87.88	93.27	86.50	83.87
MV3D [31]	LiDAR + RGB	63.51	71.29	62.68	56.56
AVOD [13]	LiDAR + RGB	75.83	84.41	74.44	68.65
3D-CVF [32]	LiDAR + RGB	82.67	89.67	79.88	78.47
EPNet [14]	LiDAR + RGB	85.00	92.28	82.59	80.14
Focals Conv [67]	LiDAR + RGB	86.84	92.26	85.32	82.95
Graph-VoI [60]	LiDAR + RGB	88.88	95.67	86.87	84.09
VirConv-L [12]	LiDAR + RGB	89.30	93.36	88.71	85.83
VPFNet [54]	LiDAR + RGB	89.41	93.42	88.76	86.05
SFD [19]	LiDAR + RGB	89.92	95.47	88.56	85.74
Bi-Att3DDet (Ours)	LiDAR + RGB	**90.49**	**95.83**	**89.10**	**86.53**

**Table 3:** Car BEV detection results on the KITTI val set with R40. Bold represents the best performance.

Method	Modality	BEV			
		mAP	Easy	Mod.	Hard
Voxel-RCNN [4]	LiDAR	91.92	95.52	91.25	88.99
DVF-PV [61]	LiDAR	92.35	96.21	91.66	89.17
SE-SSD [64]	LiDAR	92.86	96.59	92.28	89.72
MV3D [31]	LiDAR + RGB	63.51	86.02	76.90	68.49
AVOD [13]	LiDAR + RGB	75.83	86.80	85.44	77.73
3D-CVF [32]	LiDAR + RGB	88.51	93.52	89.56	82.45
EPNet [14]	LiDAR + RGB	88.79	94.22	88.47	83.69
CLOCs PVCas [15]	LiDAR + RGB	89.81	93.05	89.80	86.57
VPFNet [36]	LiDAR + RGB	92.14	94.11	92.44	89.88
SFD [19]	LiDAR + RGB	93.22	96.24	92.09	91.32
Bi-Att3DDet (Ours)	LiDAR + RGB	**93.82**	**96.71**	**92.62**	**92.13**

**Table 4:** Car 3D detection results on the KITTI val set with R11. Bold represents the best performance.

Method	Modality	$3D_{R11}$			
		mAP	Easy	Mod.	Hard
Pyramid-PV [68]	LiDAR	84.20	89.37	84.38	78.84
Voxel-RCNN [4]	LiDAR	84.29	89.41	84.52	78.93
BtcDet [65]	LiDAR	-	-	86.57	-
CasA [66]	LiDAR	85.28	89.88	86.58	79.38
Focals Conv [67]	LiDAR + RGB	86.74	89.82	85.22	85.19
VirConv-L [12]	LiDAR + RGB	-	-	86.70	-
SFD [19]	LiDAR + RGB	87.35	89.74	87.12	85.20
Bi-Att3DDet (Ours)	LiDAR + RGB	**88.04**	**90.10**	**87.89**	**86.13**

**Table 5:** Ablation study for SA layer selection.

Param	Easy	Mod.	Hard	mAP	Gain
0 *	95.47	88.56	85.74	89.92	
3	95.55	88.35	85.92	89.94	0.02
5	95.76	88.29	85.88	89.98	0.06
4	95.74	88.79	86.03	90.19	0.27

* The model without SA layers: SFD.

**Table 6:** Ablation results on the KITTI validation set by using different designed components.

Method	SARoIFE	FBIF	$3D_{R40}$			
			Easy	Mod.	Hard	mAP
(a) *	×	×	95.47	88.56	85.74	89.92
(b)	√	×	95.74	88.79	86.04	90.19
(c)	√	√	95.83	89.10	86.53	90.49
Improvement			+0.36	+0.54	+0.79	+0.57

* The model: SFD.

**Table 7:** The multi-class detection performance of our method on the KITTI val set. Bold represents the best performance.

Class	Method	$3D_{R40}$			$3D_{\mathrm{BEV}}$		
		Easy	Mod.	Hard	Easy	Mod.	Hard
Car	SFD	**95.52**	88.27	85.57	96.24	92.09	91.32
	ours	95.25	**88.32**	**85.91**	**96.29**	**92.27**	**91.85**
Pedestrian	SFD	72.94	66.69	**61.59**	75.64	69.71	64.75
	ours	**74.22**	**67.57**	61.14	**77.78**	**71.26**	**65.74**
Cyclist	SFD	93.39	72.95	67.26	93.37	75.31	70.80
	ours	**95.46**	**73.60**	**68.90**	**95.86**	**76.06**	**71.45**

**Table 8:** Ablation study on the KITTI val set with R40 for the cyclist category, demonstrating the contribution of the SARoIFE and FBIF modules.

With SARoIFE	With FBIF	$3D_{R40}$		
		Easy	Mod.	Hard
×	×	93.39	72.95	67.26
√	×	93.72 (+0.33)	73.14 (+0.19)	67.62 (+0.36)
√	√	95.46 (+1.74)	73.60 (+0.46)	68.90 (+1.28)

## Data Availability

The dataset supporting the results of this study is available on the KITTI official website: https://www.cvlibs.net/datasets/kitti/ (accessed on 10 January 2024).
